# Assessing the impact of maternal blood pressure during pregnancy on perinatal health: a wide-angled Mendelian randomization study

**DOI:** 10.1186/s12916-025-04548-3

**Published:** 2026-01-14

**Authors:** Fernanda Morales-Berstein, Ana Gonçalves-Soares, Qian Yang, Nancy McBride, Tom Bond, Marwa Al Arab, Alba Fernández-Sanlés, Maria C. Magnus, Eleanor Sanderson, Emma Hart, Abigail Fraser, Katherine A. Birchenall, Deborah A. Lawlor, Gemma L. Clayton, Maria-Carolina Borges

**Affiliations:** 1 MRC Integrative Epidemiology Unit at the University of Bristol, Augustine’s Courtyard, Orchard Lane, Bristol, BS1 5DS UK; 2 Population Health Sciences, Bristol Medical School, University of Bristol, Bristol, UK; 3 Department of Endocrine and Metabolic Diseases, Shanghai Institute of Endocrine and Metabolic Diseases, Ruijin Hospital, Shanghai Jiao Tong University School of Medicine, Shanghai, China; 4 Shanghai National Clinical Research Center for Metabolic Diseases, Shanghai Key Laboratory for Endocrine Tumor, Key Laboratory for Endocrine and Metabolic Diseases of the National Health Commission of the PR China, Lifecycle Health Management Center, Ruijin Hospital, Shanghai Jiao Tong University School of Medicine, Shanghai, China; 5 Division of Psychiatry, Faculty of Brain Sciences, University College London, London, UK; 6 Centre for Fertility and Health, Norwegian Institute of Public Health, Oslo, Norway; 7 School of Physiology, Pharmacology & Neuroscience, University of Bristol, Bristol, UK; 8 Translational Health Sciences, Bristol Medical School, University of Bristol, Bristol, UK; 9 NIHR Biomedical Research Centre, University of Bristol, Bristol, UK; 10 Department of Obstetrics and Gynaecology, St Michael’s Hospital, Bristol, BS2 8EG UK

**Keywords:** Mendelian randomization, Maternal blood pressure, Perinatal outcomes, MoBa, ALSPAC, FinnGen, BiB, UK Biobank

## Abstract

**Background:**

Observational studies link high blood pressure in pregnancy to numerous adverse pregnancy and perinatal outcomes; however, findings may be affected by residual confounding or reverse causation. This study aimed to assess the causal effect of blood pressure during pregnancy on a range of pregnancy and perinatal outcomes.

**Methods:**

We performed two-sample Mendelian randomization (MR) to assess the effect of systolic and diastolic blood pressure (SBP/DBP) during pregnancy on 16 primary and eight secondary adverse pregnancy and perinatal outcomes. We obtained genetic association data from large-scale meta-analyses of genome-wide association studies involving predominantly European ancestry individuals for SBP/DBP (*N* = 1,028,980), and pregnancy and perinatal outcomes (*N* = 74,368–714,899). We used inverse-variance weighted (IVW) MR for main analyses and MR-Egger, weighted median, weighted mode, multivariable MR, and IVW adjusted for fetal genetic effects for sensitivity analyses.

**Results:**

A 10 mmHg higher genetically predicted maternal SBP increased the odds of gestational diabetes, induction of labour, low birth weight (LBW), small-for-gestational age (SGA), preterm birth (PTB), and neonatal intensive care unit (NICU) admission (OR ranging from 1.11 [95% CI 1.02 to 1.20] for NICU admission to 1.33 [1.26 to 1.41] for LBW); while decreasing the odds of high birth weight (HBW), large-for-gestational age (LGA), and post-term birth [OR ranging from 0.76 (0.69 to 0.83) for HBW to 0.94 (0.90 to 0.99) for post-term birth]. We did not find evidence that genetically predicted higher maternal SBP was related to miscarriage or stillbirth. The results for maternal DBP were similar to the results for SBP. Overall, the main results were consistent across sensitivity analyses accounting for pleiotropic instruments and fetal genetic effects.

**Conclusions:**

Higher maternal blood pressure reduces gestation duration and fetal growth and increases the risks of induction of labour, gestational diabetes, and neonatal intensive care unit admission. This and other emerging evidence highlight the value of interventions aimed at controlling blood pressure in the population to reduce the burden of adverse pregnancy outcomes.

**Supplementary Information:**

The online version contains supplementary material available at 10.1186/s12916-025-04548-3.

## Background

High blood pressure affects approximately one in ten pregnant women, making it the most common medical problem encountered in pregnancy [1]. Hypertensive disorders of pregnancy (HDP), which include chronic hypertension, gestational hypertension (GH), and preeclampsia, are among the leading causes of adverse pregnancy and perinatal outcomes worldwide [2, 3], especially in low- and middle-income countries [4–6]. The burden of high blood pressure during pregnancy on maternal and offspring health is likely to increase with the rising prevalence of obesity and older maternal age at conception [7–9].

There is an extensive body of literature from observational studies linking higher maternal blood pressure during pregnancy, as well as HDP, with a range of adverse pregnancy and perinatal outcomes, such as stillbirth, gestational diabetes (GDM), caesarean section, induction of labour, as well as having a baby who is small-for-gestational age (SGA), preterm (PTB), or requires admission to a neonatal intensive care unit (NICU) [10–13]. The evidence from these studies indicates that preventing or treating high blood pressure during pregnancy could help avert a wide range of adverse outcomes for mothers and babies. However, such studies are likely to be influenced by residual confounding due to unmeasured or inaccurately measured characteristics, such as socioeconomic position, maternal adiposity, and comorbidities. Furthermore, most observational studies to date have examined associations with only a limited number of pregnancy or perinatal outcomes. We argue that investigating a broader spectrum of these outcomes is crucial for informing more tailored blood pressure monitoring during pregnancy and for capturing the full scope of potential benefits associated with preventing hypertensive disorders in this period.

Evidence from randomised controlled trials (RCTs) on the effect of treating mild to moderate high blood pressure during pregnancy is uncertain for many adverse pregnancy and perinatal outcomes. A Cochrane systematic review on antihypertensive treatments including 5909 women with mild to moderate hypertension during pregnancy (*N* = 58 trials) was inconclusive on the treatment benefits for reducing adverse outcomes such as fetal or neonatal death (including miscarriage) (RR = 0.72, 95% CI 0.50 to 1.04); SGA (RR = 0.96, 95% CI 0.78 to 1.18); and PTB (RR = 0.96, 95% CI 0.83 to 1.12) [14]. More recently, an open label RCT, enrolling 2408 women with mild chronic hypertension, reported that being randomised to antihypertensive medications, as opposed to no treatment (unless severe hypertension developed), decreased the risk of a composite outcome, consisting of pre-eclampsia with severe features, medically indicated PTB, placental abruption, and fetal/neonatal death [15].

Mendelian randomization (MR), a method that uses genetic variants as instrumental variables for modifiable risk factors, could help disentangle causation from confounding in an observational analysis [16, 17]. MR can strengthen causal evidence to inform clinical decisions, especially when high-quality and well-powered RCTs are unavailable. Genetic variants are randomly allocated at conception and cannot be modified by the outcome of interest. Hence, MR is less susceptible to reverse causation and confounding by factors such as socioeconomic position and related behavioural and health factors than conventional observational methods [18]. Previous MR studies have investigated the effects of higher genetically predicted maternal blood pressure on a limited set of outcomes, reporting potential effects on offspring birthweight [19, 20] and gestational duration [21, 22], although there were inconsistencies between studies, and some studies did not appropriately account for the correlation between maternal and fetal genotype.

We aimed to test the effect of higher blood pressure during pregnancy on a wider range of adverse pregnancy and perinatal outcomes. We did so by using MR to estimate the effect of genetically predicted higher systolic (SBP) and diastolic (DBP) blood pressure during pregnancy on 24 adverse maternal and offspring outcomes.

## Methods

**Fig. 1:**
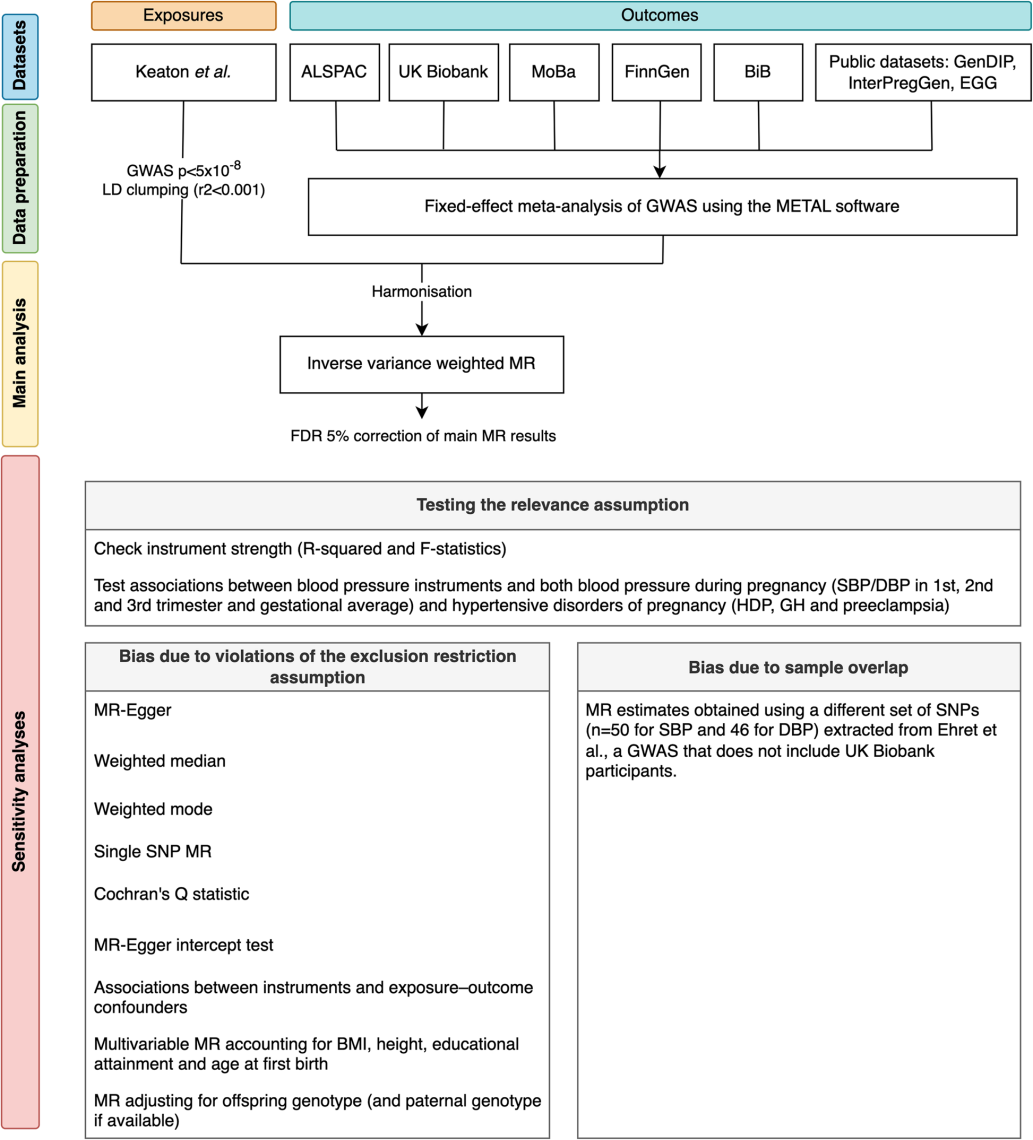
Flowchart for study design. Abbreviations: ALSPAC, Avon Longitudinal Study of Parents and Children; BiB, Born in Bradford; BMI, body mass index; DBP, diastolic blood pressure; EGG, Early Growth Genetics consortium; FDR, false discovery rate; GenDIP, Genetics of Diabetes in Pregnancy consortium; GH, gestational hypertension; GWAS, genome-wide association study; HDP, hypertensive disorders of pregnancy; InterPregGen, International Pregnancy Genetics study; LD, linkage disequilibrium; MoBa, Norwegian Mother, Father and Child Cohort Study; MR, Mendelian randomization; PGC, Psychiatric Genomics Consortium; SBP, systolic blood pressure. Genetic association data for SBP and DBP were obtained from Keaton et al. [23] (main analysis) and Ehret et al. [26] (sensitivity analysis to assess bias due to sample overlap)

We examined the effect of maternal genetically predicted blood pressure on 16 primary and eight secondary pregnancy and perinatal outcomes using a two-sample MR framework (Fig. 1). First, we selected genetic instruments for SBP (545 single-nucleotide polymorphisms (SNPs)) and DBP (513 SNPs) identified in a genome-wide association study (GWAS) meta-analysis by Keaton et al. [23], including 1,028,980 European non-pregnant women and men (sample 1). Then, we extracted pregnancy and perinatal outcomes GWAS data for the same SNPs from up to 714,899 women from the MR-PREG collaboration [24] (sample 2). Genetic association data from samples 1 and 2 were used to estimate the effect of maternal genetically predicted blood pressure (i.e. SBP and DBP) on the 24 pregnancy and perinatal outcomes. We conducted a series of sensitivity analyses to explore the plausibility of the core MR assumptions as outlined in Fig. 1 and detailed under “sensitivity analyses”. This study was reported following the recommendations of the Strengthening the Reporting of Observational Studies in Epidemiology using Mendelian Randomization (STROBE-MR) guidelines [25] (Additional File 1).

### Data sources

Details regarding the generation of genetic association data for blood pressure traits, perinatal outcomes, and data used in sensitivity analyses are provided in Additional File 2: Supplementary Methods [24, 27–43].

We used genetic association data for SBP and DBP from the GWAS meta-analyses by Keaton et al. [23] to maximise statistical power given this was the largest data source available at the time of the study (*N* = 1,028,980 European women and men). For sensitivity analyses assessing bias due to sample overlap, we also selected SNPs from Ehret et al. [26], a GWAS meta-analysis including 201,529 European women and men, who are not part of UK Biobank (UKB). In both GWAS, SBP and DBP were measured using standard protocols detailed elsewhere [23, 26]. For individuals treated with antihypertensive medication, measured values were adjusted by adding 15 and 10 mmHg to SBP and DBP, respectively, to account for antihypertensive treatment effects that could lead to the underestimation of the GWAS estimates [23]. Table 1 summarises key information on the GWAS used for SBP and DBP phenotypes.

**Table 1:** Description of publicly available GWAS used for SBP and DBP phenotypes

Study	Consortium (PMID)	Units	Sample size, *n*	European, %	UKB female participants, %	Sex	Covariate adjustments ^a^	Accession codes
Keaton [23]	UKB + ICBP + MVP + BioVU (38,689,001)	mmHg	1,028,980	100%	24%	Male and female	Sex, age, age^2^ and BMI	GCST90310294 (SBP) [44] GCST90310295 (DBP) [45]
Ehret [26]	ICBP (27,618,452)	mmHg	201,529	100%	0%	Male and female	Sex, age, age^2^ and BMI	GCST006259 (SBP) [46] GCST006258 (DBP) [47]

^a^Additional adjustments: UKB was adjusted for genotyping chips. ICBP studies were optionally adjusted for population stratification using different methods (e.g. adjustment for principal components, adjustment for study site, multidimensional scaling). For more details, see Keaton et al. [23] and Ehret et al. [26]. These data were downloaded from the NHGRI-EBI GWAS Catalog [48] data repository using the “MRInstruments” R package using the codes listed under the “Accession codes” column

Abbreviations: UKB UK Biobank, ICBP International Consortium for Blood Pressure, MVP Million Veteran Program, BioVU Vanderbilt University’s biorepository of DNA linked to de-identified medical records, SBP systolic blood pressure, DBP diastolic blood pressure, BMI body mass index

We were interested in 16 primary and eight secondary outcomes. Outcomes were classified as secondary if they were a subtype of or the continuous trait underlying a primary outcome. Our outcomes of interest included the following: (1) *pregnancy loss outcomes* including miscarriage (with sporadic and recurrent miscarriage as secondary outcomes) and stillbirth; (*2*) *maternal morbidity outcomes* including GDM and perinatal depression; (3) *labour outcomes* including induction of labour, pre-labour rupture of membranes, and caesarean section (c-section, with emergency and elective c-section as secondary outcomes); (4) *offspring birth outcomes* including low and high birth weight (LBW/HBW) (with continuous birth weight in standard deviations as a secondary outcome); small- and large-for-gestational age (SGA/LGA), PTB and post-term birth (with continuous gestational age in weeks, and very PTB and spontaneous PTB as secondary outcomes); low Apgar score at 1 and 5 min, and NICU admission. Details on the outcome definitions can be found in Additional File 3: Supplementary Tables 1A–C.

Outcome data were obtained from the MR-PREG collaboration [24], which at the time of this study, included data from three birth cohorts—i.e. the Avon Longitudinal Study of Parents and Children (ALSPAC) [27, 28], Born in Bradford (BiB) [29], and the Norwegian Mother, Father and Child Cohort Study (MoBa) [37, 38]; two biobanks—i.e. UKB [33, 34] and FinnGen [39]; and four GWAS meta-analyses—i.e. the Early Growth Genetics (EGG) consortium [41], the International Pregnancy Genetics study (InterPregGen) [42], the Genetics of Diabetes in Pregnancy (GenDIP) consortium [40], and the Psychiatric Genomics Consortium (PGC) [43].

ALSPAC is a prospective birth cohort that recruited pregnant women from the former county of Avon, UK, between April 1991 and December 1992, enrolling 14,541 women and following their children over time [27, 28]. The study collected extensive questionnaire data, biological samples, and anthropometric measurements, with genetic data available for mothers, partners, and children.

BiB is a prospective birth cohort that recruited 12,453 pregnant women in Bradford, UK, between 2007 and 2010, with most enrolled during their oral glucose tolerance test at 26–28 weeks of gestation [29]. The study collected biological samples, anthropometric data, and questionnaire responses, with linkage to primary and secondary care records and genetic data available for mothers and children.

MoBa is a nationwide Norwegian birth cohort that recruited over 95,200 mothers and 75,200 fathers between 1999 and 2008, with follow-up of 114,500 children [37, 38]. The study collected questionnaire data, biological samples, and anthropometric measures, with linkage to the national Medical Birth Registry and genetic data available for families.

UKB is a large-scale adult cohort study that recruited 500,000 individuals aged 40–69 years across the UK between 2006 and 2010 [33, 34]. The study collected extensive baseline data, biological samples, and health measures, with ongoing follow-up via electronic health records and linkage to hospital and maternity admissions data.

FinnGen [39] is a nationwide Finnish biobank network that integrates genetic data from 500,348 individuals with national electronic health registries, providing detailed information on prescriptions and disease diagnoses (ICD-9, ICD-10 codes). The study includes clinical endpoints, including adverse pregnancy and perinatal outcomes, with case–control data available.

Where external, quality-controlled GWAS meta-analysis data from key genetic consortia were available, the MR-PREG collaboration [24] harmonised these to supplement the sample size for the following outcomes: GDM from GenDIP (5569 cases, 353,157 controls) [40]; preeclampsia from InterPregGen (7219 cases, 155,660 controls) [42]; gestational duration traits from EGG (including PTB, post-term birth, and gestational age at delivery) [41]; and postnatal depression from PGC (13,348 cases, 41,127 controls) [43].

### Data analyses

#### Genetic instrument selection

We selected genetic instruments from the Keaton et al. [23] GWAS to maximise statistical power. GWAS-significant SNPs (*p* < 5 × 10^−8^) were selected as candidate instrumental variables for SBP and DBP. Among correlated SNPs, those with the smallest *p*-values were identified and retained via linkage disequilibrium (LD) clumping (*r*^2^ < 0.001, window size of 10,000 kb), using the 1000 Genomes European ancestry reference panel.

#### Genetic instrument relevance

We estimated instrument strength in the original SBP/DBP GWAS meta-analyses by calculating the F-statistic and R^2^ for the selected SNPs as follows: ${R}^{2} (2{\beta }_{SD}^{2}\times MAF\times (1-MAF))$ [49] and $F= \frac{{\beta }^{2}}{{se}^{2}}$ [50], where MAF is the minor allele frequency, N is the sample size, and β is the effect estimate of the instrumental variable—blood pressure association with corresponding standard error (SE). We then computed the mean and range of the F-statistics, as well as the total R^2^ for each set of SBP/DBP instruments.

Due to the absence of large-scale GWAS meta-analyses of SBP and DBP measured during pregnancy, we selected SNPs from GWAS meta-analyses of non-pregnant individuals. This assumes that these SNPs are strong instruments for the exposure of interest, i.e. mean SBP and DBP during pregnancy. We tested this assumption in two steps.

First, we compared the SNP effects on SBP/DBP that were obtained using measurements of blood pressure taken among the general population (in mmHg) [23] with those obtained using measurements taken during the first, second, and third trimesters, and across gestation (in mmHg using ALSPAC data). We calculated Pearson correlation coefficients to quantify these relationships. In addition, we ran linear regression models with SNP effect estimates from the general population as the independent variable and during pregnancy as the dependent variable. From these models, we extracted the regression intercept, slope, and R^2^. A slope of 1 implies that the SNPs have the same average effect in both ALSPAC and the general population; a slope < 1 implies smaller effects in ALSPAC while a slope > 1 implies larger effects in ALSPAC.

Second, we checked whether there was a positive correlation between the SNP effects on SBP/DBP in the general population (from Keaton et al. [23]) and the SNP effects on the risk of HDP (cases/*N* = 32,549/541,768); GH (cases/*N* = 20,777/527,932); and preeclampsia (cases/*N* = 19,408/671,992) among the participants included in the MR-PREG meta-analyses.

#### Data harmonisation

Genetic association data for SBP and DBP were harmonised with those for perinatal outcomes to ensure effect alleles matched across datasets. Positive strands of palindromes were aligned using minor allele frequencies (MAF). Palindromic SNPs with MAF ≥ 0.42 or strand mismatch were excluded.

#### MR analyses

##### Main analyses

The main MR analyses were performed using the inverse-variance weighted (IVW) method [51], which has the greatest statistical power. MR estimates were scaled to reflect a 10-mmHg increase in genetically predicted SBP and DBP (this was also done in the sensitivity MR analyses described below).

A 5% Benjamini–Hochberg False Discovery Rate (FDR) correction [52] was applied to all main IVW results to account for multiple testing. Furthermore, we used the Steiger directionality test to assess whether the SNPs used as instruments for maternal blood pressure in the main analyses explained more variance in the exposures (SBP/DBP) than in the outcomes [53]. Evidence that the SNPs account for more variance in the exposures than in the outcomes would support a causal effect in the direction of maternal blood pressure influencing the perinatal outcomes. Conversely, if the SNPs explain more variance in an outcome than in the exposures, it would suggest a potential reverse causal effect, whereby the perinatal outcome may influence maternal blood pressure.

##### Sensitivity analyses

We conducted a series of sensitivity analyses to assess the robustness of our main MR estimates. These were mainly focused on assessing the plausibility of the core assumptions required for MR to reliably test the presence of a causal effect: genetic instruments must (1) be strongly statistically associated with blood pressure during pregnancy, (2) share no common cause with the perinatal outcomes of interest, and (3) only be associated with the perinatal outcomes via their effect on maternal blood pressure (e.g. no unbalanced horizontal pleiotropy) [54]. These are generally referred to as the relevance, independence, and exclusion restriction assumptions, respectively. We tested the relevance assumption as described above under “Genetic instrument relevance”. As an additional check, we conducted MR analyses assessing the effects of genetically predicted SBP/DBP on positive control outcomes (i.e. stroke and coronary artery disease). Genetic association data for these outcomes were accessed through the IEU OpenGWAS database API (IDs: ebi-a-GCST005838 and ebi-a-GCST003116, respectively) [55, 56]. For the independence and exclusion restriction assumptions, we assessed their plausibility through the sensitivity analyses described below.

- Bias due to violations of the independence assumption.

The independence assumption of no confounding between the genetic instrument and outcomes could be violated by population stratification, assortative mating, or dynastic effects [57]. For biological traits such as blood pressure, assortative mating and dynastic effects are less likely to be key sources of bias [58]. To mitigate bias due to population stratification, all GWASs used in our analyses were adjusted for principal components of ancestry and/or used mixed models.

b) Bias due to violations of the exclusion restriction assumption

We reran the analyses using MR methods that are understood to be more robust in the presence of horizontal pleiotropy: MR-Egger [59], weighted median [60], and weighted mode [61] methods. All of these rely on different assumptions about the nature of any pleiotropic effects. MR-Egger assumes instrument-outcome pleiotropic effects are independent of instrument-risk factor associations (Instrument Strength Independent of Direct Effect—InSIDE—assumption). The slope of the MR-Egger regression represents the estimate of the true causal effect, if the InSIDE [59] assumption holds. The weighted median estimator [60] assumes that at least 50% of the weight in the analyses comes from valid instruments. The weighted mode estimator assumes that the most frequent effect equals the true causal effect (ZEro Modal Pleiotropy Assumption—ZEMPA) [61]. Consistent results across methods are supportive of MR estimates being unbiased.

Cochran’s Q statistic was used to quantify heterogeneity across estimates obtained from different SNPs [62]. A large Q statistic is indicative of high levels of heterogeneity in the individual SNP estimates, possibly but not necessarily due to horizontal pleiotropy. Importantly, this test is not specific to violations of the exclusion restriction criteria but can indicate that one or more genetic variants violate the MR assumptions. Additionally, MR-Egger intercept tests were performed to assess statistical evidence for unbalanced horizontal pleiotropy. A non-zero intercept (*p* < 0.05) can be interpreted as evidence of unbalanced pleiotropic bias.

To investigate potential specific pleiotropic pathways (via established risk factors for adverse perinatal outcomes that are unlikely to be affected by maternal blood pressure), we also conducted MR analyses assessing the association of genetically predicted SBP and DBP with height (id: ieu-b-4814) [63]; body mass index (BMI; id: ieu-b-4816) [64], age at first birth (id: ieu-b-4820) [65]; age at menarche (id: ieu-b-4822) [66]; cigarettes smoked per day (id: ieu-b-4826) [67]; number of children ever born (id: ieu-b-4828) [68]; alcohol consumption (id: ieu-b-4834) [69]; years of schooling (id: ieu-b-4836) [70]; and physical activity (id: ieu-b-4860) [71] in the UKB [72]. Details of how these risk factors were assessed are provided in Additional File 2: Supplementary Methods. Any evidence of an effect would suggest potential violations of the exclusion restriction assumption. We used multivariable MR [73] to account for any potentially pleiotropic risk factors (i.e. that were predicted by our genetic instruments for SBP/DBP) by including them as additional exposures in each genetically instrumented maternal blood pressure-perinatal outcome model. The multivariable MR exposure and outcome datasets were all harmonised to the same effect allele, and we calculated the conditional F-statistics to test whether the main exposure could be strongly predicted in the multivariable MR. Given the partial sample overlap between some of the exposure GWASs in our model, we approximated the pairwise covariances between SNP–exposure associations using the phenotypic correlations between exposures in UKB, as recommended by Sanderson et al. [74].

Furthermore, MR analyses adjusted for offspring genotype were conducted to investigate whether MR estimates were biased by fetal effects of maternally inherited alleles (which would lead to the violation of the exclusion restriction assumption). We accounted for a potential effect from fetal genetic variants using a weighted linear model (WLM) [75, 76]. First, we used a WLM to estimate conditional genetic effects—i.e. maternal genetic effects on outcomes adjusted by offspring genotype. Second, we used IVW, as in the main analyses, with conditional estimates for maternal genetic effects on each outcome. We conducted additional analyses in which we simultaneously adjusted for offspring and paternal genetic genotypes, whenever available, due to the potential collider bias related to only conditioning on fetal genotype [77].

c) Bias due to sample overlap

Bias due to sample overlap between exposure and outcome datasets was assessed by comparing IVW MR estimates from partially overlapping datasets (Keaton et al. [23]) with those from non-overlapping datasets (Ehret et al. [26]).

#### Software

All analyses were conducted using R version 4.4.0. Two-sample MR analyses were performed using the “TwoSampleMR” package version 0.6.6. Multivariable MR analyses were conducted using the “MVMR” package version 0.4 available at https://github.com/WSpiller/MVMR. The WLM models were implemented in the DONUTS R package version 1.0.0 [76]. Plots were created using “ggplot2” package version 3.5.1 and the “ggforestplot” package version 0.1.0.

## Results

### Descriptive results

The total number of participants (and cases) for each outcome included in the present MR study is shown in Table 2. The number of participants ranged from 74,368 (low Apgar score at 5 min) to 714,899 (GDM), while the number of cases ranged from 821 (low Apgar score at 5 min) to 89,086 (miscarriage).

**Table 2:** Total number of participants included in the study by outcome of interest

Outcome	Type	Cases (*N*)	Cases (%)	*N*
**Pregnancy loss outcomes**				
Miscarriage	Primary	89,086	18%	486,217
Stillbirth	Primary	6331	3%	207,670
Sporadic miscarriage	Secondary	55,888	23%	241,159
Recurrent miscarriage	Secondary	6233	2%	321,756
**Maternal morbidity outcomes**				
Gestational diabetes	Primary	24,752	3%	714,899
Perinatal depression	Primary	17,076	15%	115,797
**Labour outcomes**				
Induction of labour	Primary	14,495	15%	95,239
Rupture of membranes	Primary	21,839	7%	310,621
Caesarean section	Primary	33,909	14%	233,969
Emergency C-section	Secondary	9165	10%	90,438
Elective C-section	Secondary	6004	7%	87,283
**Offspring birth outcomes**				
Low birth weight	Primary	19,180	7%	283,151
High birth weight	Primary	6679	3%	266,835
Small for gestational age	Primary	7448	8%	96,305
Large for gestational age	Primary	10,468	11%	96,305
Preterm birth	Primary	18,225	6%	285,722
Post-term birth	Primary	27,213	7%	414,667
Low Apgar score at 1 min	Primary	4950	6%	86,668
Low Apgar score at 5 min	Primary	821	1%	74,368
NICU admission	Primary	6996	9%	77,285
Spontaneous preterm birth	Secondary	17,176	6%	272,940
Very preterm birth	Secondary	1107	1%	77,683
Birth weight	Secondary	NA	NA	289,846
Gestational age	Secondary	NA	NA	211,416

Abbreviations: *NICU* neonatal intensive care unit, *NA* not applicable, *N* maximum number of participants (it may be lower for some genetic variants)

#### Genetic instruments relevance

We selected 545 and 513 independent GWAS-significant SNPs (*p* < 5 × 10^−8^) as instrumental variables for SBP and DBP, respectively. Altogether, they each explained 3.5% and 4.4% of the variance in SBP and DBP. Mean F-statistics across SNPs corresponded to 80 and 82 for the SBP and DBP instruments, respectively. Genetic instruments used in the analyses are detailed in Additional File 3: Additional File Supplementary Tables 2–5.

The selected SNPs were associated with SBP/DBP during pregnancy and with the risk of HDP, GH, and preeclampsia (correlations are shown in Additional File 4: Supplementary Figs. 1A–E and the regression intercept, slope, and R^2^ are shown in Additional File 4: Supplementary Figs. 2A–E). For illustration, there was a positive correlation between the effect of the blood pressure increasing alleles on SBP in the general population and their effect on SBP among study participants in their first (Pearson’s coefficient (*r*) = 0.34, *p* = 4.4 × 10^−16^); second (*r* = 0.39, *p* = 2.2 × 10^−16^); and third trimesters of pregnancy (*r* = 0.39, *p* = 2.2 × 10^−16^), as well as across their full gestation (*r* = 0.42, *p* = 2.2 × 10^−16^). Corresponding correlations for DBP ranged from 0.19 (*p* = 1.3 × 10^−5^) to 0.49 (*p* = 2.2 × 10^−16^). Similarly, the regression models to examine the association of SNPs with SBP and DBP throughout the gestational period had, respectively, intercepts of − 0.01 and 0.01, and slopes of 0.34 (*p* = 9.7 × 10^−25^) and 0.45 (*p* = 1.9 × 10^−30^). In addition, there was a positive correlation between the effect of blood pressure increasing alleles on SBP in the general population and their effect on the risk of HDP (*r* = 0.76, *p* < 2.2 × 10^−16^); GH (*r* = 0.75, *p* < 2.2 × 10^−16^); and preeclampsia (*r* = 0.64, *p* < 2.2 × 10^−16^). Corresponding correlations for DBP ranged from 0.64 (*p* < 2.2 × 10^−16^) to 0.73 (*p* < 2.2 × 10^−16^).

#### Main Mendelian randomization results for primary outcomes

**Fig. 2:**
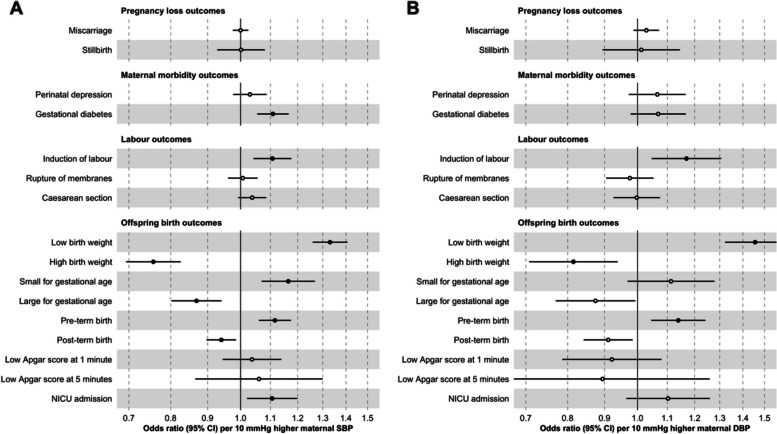
Inverse-variance weighted Mendelian randomization estimates for genetically predicted effects of maternal blood pressure on the primary outcomes. Odds ratios and 95% confidence intervals are presented per 10 mmHg increase in systolic (**A**) and diastolic (**B**) blood pressure. Systolic and diastolic blood pressure were instrumented by 545 and 513 genetic variants obtained from Keaton et al. [23], respectively. Estimates with a false discovery rate corrected *p*-value < 0.05 are indicated by filled black circles.

Higher genetically instrumented maternal SBP was related to higher odds of GDM (OR = 1.11, 95% CI 1.05 to 1.17, per 10 mmHg); induction of labour (OR = 1.11, 95% CI 1.04 to 1.18); LBW (OR = 1.33, 95% CI 1.26 to 1.41); SGA (OR = 1.16, 95% CI 1.07 to 1.27); PTB (OR = 1.12, 95% CI 1.06 to 1.17); and NICU admission (OR = 1.11, 95% CI 1.02 to 1.20) and lower odds of HBW (OR = 0.76, 95% CI 0.69 to 0.83); LGA (OR = 0.87, 95% CI 0.80 to 0.94); and post-term birth (OR = 0.94, 95% CI 0.90 to 0.99). All these results passed our criteria for multiple testing correction. We did not find evidence that higher maternal SBP was related to miscarriage (OR = 1.00, 95% CI 0.98 to 1.02) or stillbirth (OR = 1.00, 95% CI 0.93 to 1.08). For other outcomes, the results were inconclusive due to the modest magnitude and relatively high imprecision of the effect estimates—i.e. perinatal depression (OR = 1.03, 95% CI 0.98 to 1.09) and low Apgar at 1 (OR = 1.04, 95% CI 0.94 to 1.14) and 5 min (OR = 1.06, 95% CI 0.87 to 1.30) (Fig. 2A and Additional File 3: Additional File Supplementary Table 6). The results for maternal DBP were broadly similar to the results for SBP, although they were estimated with higher imprecision (Fig. 2B and Additional File 3: Additional File Supplementary Table 6). Furthermore, we confirmed that the SNPs used as genetic instruments for blood pressure primarily influenced the exposures (SBP/DBP) rather than the perinatal outcomes (Additional File 3: Supplementary Table 7). Study-specific estimates are displayed in Additional File 4: Supplementary Fig. 3.

### Main Mendelian randomization results for secondary outcomes

Consistent with findings from the primary outcomes, higher maternal SBP was related to secondary outcomes in a similar pattern: increased odds of emergency and elective C-section, spontaneous and very PTB, but not pregnancy loss outcomes (sporadic and recurrent miscarriage) (Additional File 4: Supplementary Fig. 4A and Additional File 3: Supplementary Table 6). Additionally, higher SBP was linked to lower birthweight and shorter gestational period (Additional File 4: Supplementary Fig. 5A and Additional File 3: Supplementary Table 6). Higher maternal DBP was associated with increased odds of adverse labour outcomes (elective C-section only) and offspring outcomes (spontaneous and very PTB, lower birthweight, and shorter gestational age) (Additional File 4: Supplementary Figs. 4B and 5B and Additional File 3: Supplementary Table 6). In contrast to the main results regarding any/sporadic miscarriage, higher DBP was related to higher odds of recurrent miscarriage (Additional File 4: Supplementary Fig. 4B and Additional File 3: Supplementary Table 6). Study-specific estimates are displayed in Additional File 4: Supplementary Figs. 6–7.

### Sensitivity analyses

There was some evidence of heterogeneity between SNP estimates for 12/16 primary outcomes in SBP analyses and 11/16 primary outcomes in DBP analyses (Cochran’s *Q* = 502 to 783, *p* < 0.001) (Additional File 3: Supplementary Table 8).

We found evidence that a higher genetically predicted SBP/DBP increased the risk of positive control outcomes (i.e. stroke and coronary artery disease) (Additional File 4: Supplementary Fig. 8).

The MR-Egger intercept test suggested that there was detectable evidence of unbalanced horizontal pleiotropy for SBP with the odds of induction of labour (intercept, − 0.004; *p* = 0.02) and low Apgar score at 5 min (intercept, − 0.014; *p* = 0.03). For DBP, there was detectable evidence with LGA (intercept, 0.007; p = 0.008); GDM (intercept, 0.005; *p* = 0.01); HBW (intercept, 0.007; *p* = 0.02); and low Apgar score at 5 min (intercept, − 0.014; *p* = 0.04) (Additional File 3: Additional File Supplementary Table 9).

**Fig. 3:**
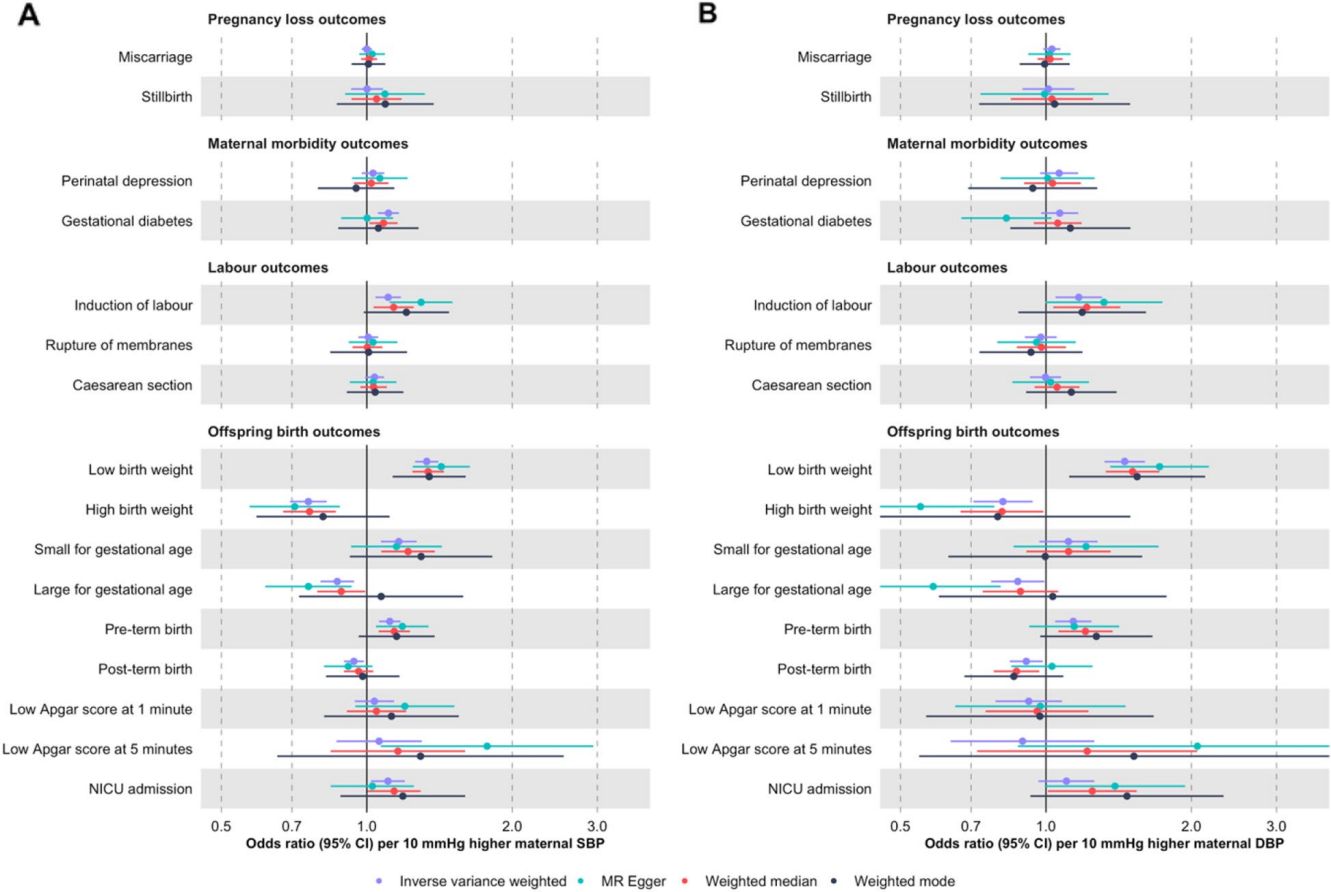
Mendelian randomization estimates for genetically predicted effects of maternal blood pressure on the primary outcomes across different methods. Odds ratios and 95% confidence intervals are presented per 10 mmHg increase in systolic (**A**) and diastolic (**B**) blood pressure. Systolic and diastolic blood pressure were instrumented by 545 and 513 genetic variants obtained from Keaton et al. [23], respectively.

As observed in Fig. 3 (primary outcomes) and Additional File 4: Supplementary Figs. 9 − 10 (secondary outcomes), the direction of the MR estimates from the main analyses using IVW was consistent with the direction of the MR estimates in sensitivity analyses exploring potential bias due to unbalanced pleiotropy using MR-Egger, weighted median, and weighted mode methods (Additional File 3: Supplementary Table 6). In some instances, the MR-Egger and/or the weighted mode estimates were attenuated and had larger confidence intervals (e.g. GDM, LGA, PTB, post-term birth, and NICU admission).

**Fig. 4:**
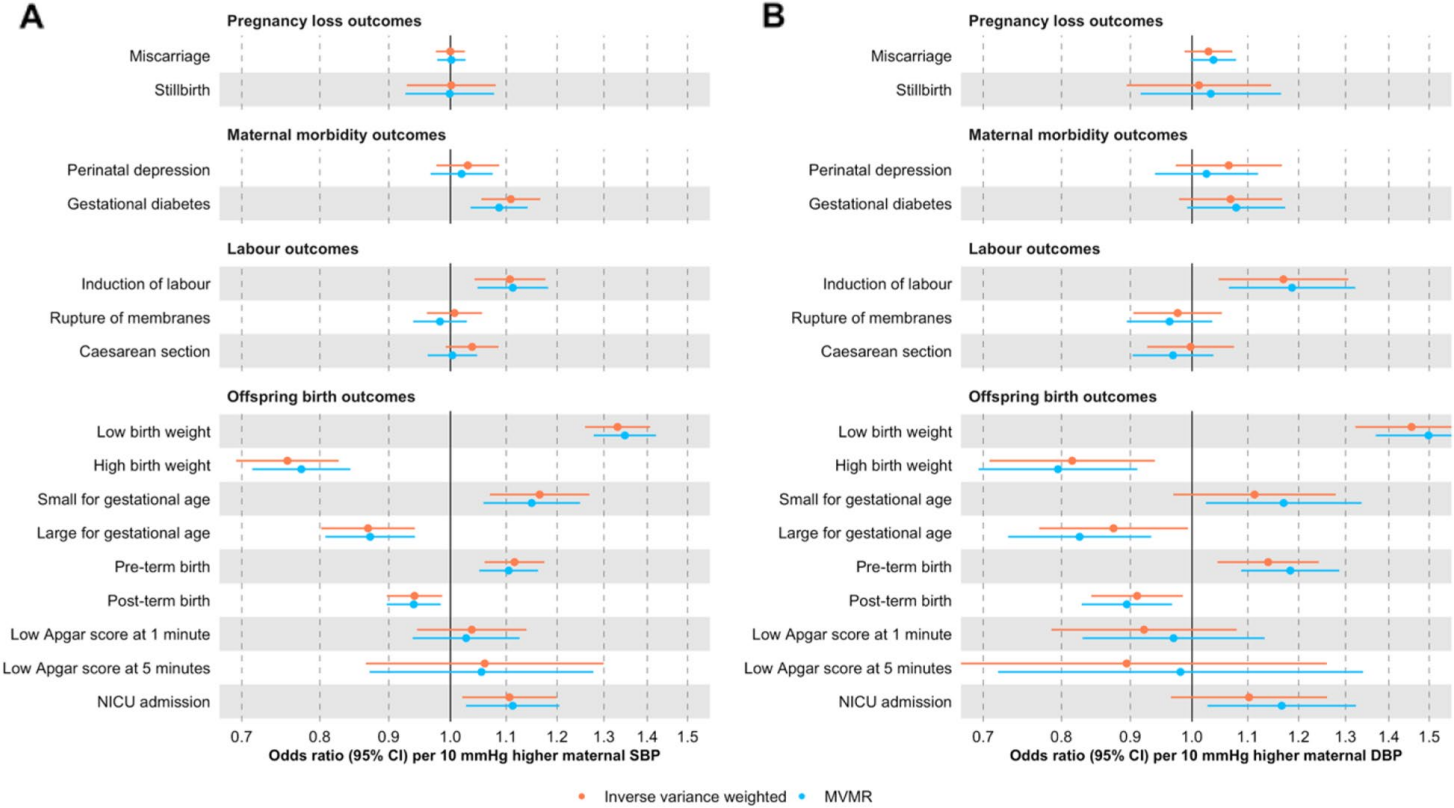
Univariable (“inverse variance weighted”) and multivariable (“MVMR”) Mendelian randomization estimates for genetically predicted effects of maternal blood pressure on the primary outcomes. Multivariable models have been adjusted for body mass index, height, years of education, and age at first birth. Odds ratios and 95% confidence intervals are presented per 10 mmHg increase in systolic (**A**) and diastolic (**B**) blood pressure. Systolic and diastolic blood pressure were instrumented by 545 and 513 genetic variants obtained from Keaton et al. [23], respectively.

We further investigated potential bias due to horizontal pleiotropy. First, we checked whether higher blood pressure, as instrumented by our selected SNPs, affected well-established determinants of adverse pregnancy/perinatal outcomes. We found that higher genetically predicted blood pressure, particularly SBP, was associated with lower height, BMI, younger age at first birth, and fewer years of schooling (Additional File 4: Supplementary Fig. 11). The association of blood pressure increasing alleles with lower BMI is likely explained by the use of BMI-adjusted GWAS summary statistics for SBP/DBP, which has been shown to potentially lead to collider stratification bias in MR [78, 79]. Second, we used multivariable MR to account for putative colliders (BMI) and pleiotropic factors (height, younger age at first birth, and fewer years of schooling). We found that the direction of the effect estimates remained unchanged and that their magnitude was only marginally affected (Fig. 4, Additional File 4: Supplementary Figs. 12 − 13, Additional File 3: Supplementary Table 10). The conditional F-statistics for SBP and DBP in the multivariable MR analyses ranged from 15 to 34, suggesting that it is unlikely that the results were affected by substantial weak instrument bias.

**Fig. 5:**
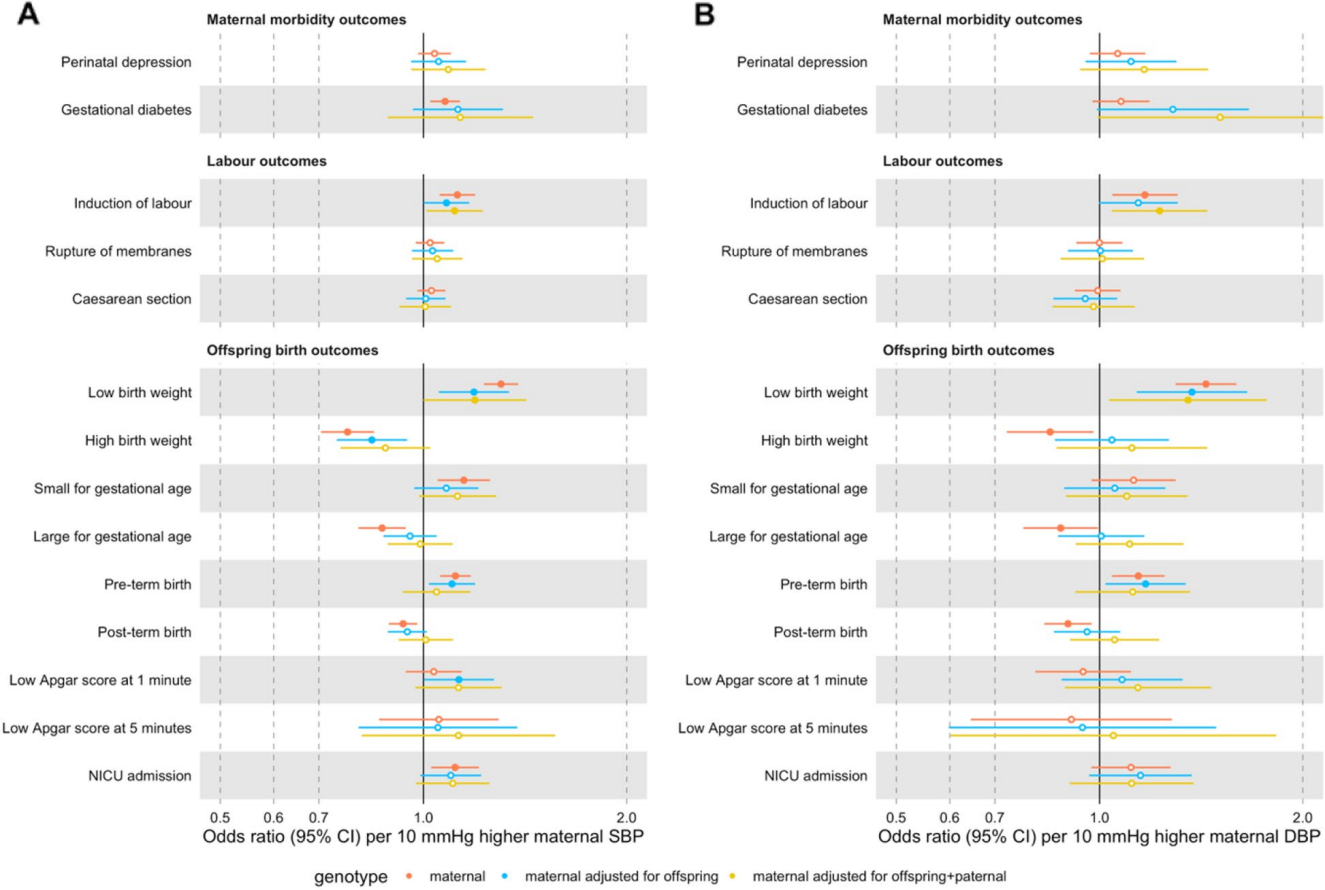
Mendelian randomization estimates for genetically predicted effects of maternal blood pressure on the primary outcomes, adjusting for offspring genotype. Odds ratios and 95% confidence intervals are presented per 10 mmHg increase in systolic (**A**) and diastolic (**B**) blood pressure. Estimates are shown for maternal effects unadjusted (red) and adjusted by fetal genetic effects (blue), and adjusted for both offspring and. genetic effects (yellow). Systolic and diastolic blood pressure were instrumented by 545 and 513 genetic variants obtained from Keaton et al. [23], respectively. Only studies with available offspring genetic data contributed to estimating fetal genetic effects (i.e. ALSPAC, BiB, MoBa studies and the EGG consortium). Abbreviations: SBP, systolic blood pressure; DBP, diastolic blood pressure; NICU, neonatal intensive care unit

Additionally, in intergenerational analyses such as the present study, there could be bias by fetal genetic effects—i.e. where fetal genotype, inherited from the mother, affects the outcomes of interest. To test whether this was likely to be a source of bias in our main findings, we conducted MR analyses that accounted for offspring genotype. The direction of the effect estimates accounting for offspring genotype was generally consistent with the direction of the effect estimates in the main analysis, both for primary (Fig. 5 and Additional File 3: Supplementary Table 11) and secondary outcomes (Additional File 4: Supplementary Figs. 14–15). For outcomes related to fetal growth (e.g. SGA and LGA), adjusting for fetal genetic effects led to the partial attenuation of effect estimates (Fig. 5, Additional File 4: Supplementary Fig. 15 and Additional File 3: Supplementary Table 11).

Finally, we did not observe any evidence of bias due to sample overlap; the results from the non-overlapping samples (Ehret et al. [26] sensitivity analyses) are consistent with those from the overlapping samples (Keaton et al. [23] main analyses) (Additional File 4: Supplementary Figs. 16–18 and Additional File 3: Supplementary Table 6).

## Discussion

Our findings align with a substantial body of observational research suggesting that elevated blood pressure during pregnancy impairs fetal growth and shortens gestational duration [10, 11, 13]. The estimated effect of higher blood pressure on the risk of PTB may be partly explained by hypertension being a common reason for induction and medically-indicated PTB. However, it is important to note that we also observed a potential effect of higher maternal blood pressure on the risk of spontaneous PTB. Additionally, our results add to existing evidence that elevated blood pressure during pregnancy may increase the risk of GDM and NICU admission. Nevertheless, our results contrast with those from previous observational studies, which found that higher blood pressure during pregnancy increases the risk of miscarriage and stillbirth [10, 13].

During pregnancy, blood pressure initially declines, before progressively increasing towards term. In a study including 4607 women with low-risk pregnancies, median SBP was lowest at 12 weeks (111.5 mmHg, 95% CI 111.3 to 111.8), rising to maximum at 40 weeks of gestation (119.6 mmHg, 95% CI 118.9 to 120.3). Median DBP was lowest at 19 weeks of gestation (68.5 mmHg, 95% CI 68.3 to 68.7) and peaked at 40 weeks of gestation (76.3 mmHg, 95% CI 75.9 to 76.8) [80]. Normal ranges for blood pressure not only vary with gestational age but also by maternal characteristics (nulliparous vs multiparous, pre-pregnancy BMI, and smoking status) [81]. This early decline in blood pressure reflects a normal physiological adaptation to pregnancy, driven by a reduction in systemic vascular resistance [82]. This reduction is primarily due to the development of low resistance in uteroplacental circulation and hormonal influences, including the increased production of vasodilatory hormones such as relaxin [82]. Based on the expected decline in blood pressure during the first 20 weeks of gestation, evidence of hypertension before this period is classified as chronic hypertension and after this period as new-onset hypertension (i.e. gestational hypertension or preeclampsia) [83]. Despite the dynamic nature of blood pressure across gestation, we showed that our selected genetic instruments were associated with similar differences in SBP and DBP across trimesters.

Existing evidence indicates that greater increases in maternal blood pressure from early (18 weeks) to late (36 weeks) pregnancy are related to reduced fetal growth and shorter gestation, even among women who remained normotensive throughout pregnancy and did not meet the criteria for HDP [84]. These associations are plausibly mediated by impaired oxygen and nutrient delivery to the fetus [85], as elevated blood pressure can disrupt key physiological processes at various stages of pregnancy, e.g. early placentation and subsequent placental perfusion [86]. Interestingly, a previous study using haplotype-based genetic scores suggested that maternal blood pressure effects on offspring birthweight were biased by offspring genetic effects [22]. In our study, we observed only partial attenuations when accounting for offspring genetic effects, indicating that maternal blood pressure has a causal effect on offspring birthweight, as corroborated by other studies [20]. Our findings also suggest that higher genetically predicted maternal blood pressure is associated with an increased risk of labour induction, as expected given that HDP are a known indication for induction [87]. Additionally, we observed a higher risk of NICU admission, with prior research identifying PTB, respiratory disease, and hypoglycaemia as the most common primary reasons for admission linked to maternal hypertension [88].

Unlike conventional observational studies [10, 13], we were unable to confirm an effect of maternal blood pressure on miscarriage and stillbirth, despite observing an impact on fetal growth restriction and preeclampsia. Two plausible explanations for these findings are that previous studies may have overestimated the effect due to residual confounding (e.g. by factors that may independently affect maternal blood pressure and stillbirth, such as smoking or placental factors), or that effects are only present at more extreme values of blood pressure which may not be adequately captured by our genetic instruments since these generally explain only a small proportion of blood pressure variation.

Our observation that higher blood pressure during pregnancy was related to higher risk of GDM is in line with conventional observational and MR studies’ finding of a positive link between blood pressure and the risk of type 2 diabetes. However, it is uncertain whether these findings reflect a true causal effect of blood pressure—potentially through microvascular damage which results in insulin resistance [89]—or whether they are explained by pleiotropic mechanisms influencing both blood pressure and glucose regulation, which is somewhat supported by the attenuated effect estimates from some of the MR methods that are more robust to horizontal pleiotropy.

Overall, our findings were more precisely estimated for SBP than DBP. Given that the selected genetic variants explain a similar amount of variance for SBP and DBP throughout pregnancy, these differences in precision cannot be explained by variation in the strength of the SBP and DBP instruments. We speculate that this might reflect different mechanisms by which higher SBP and DBP impact pregnancy outcomes. For example, high SBP predominantly causes microvascular damage and vessel/cardiac remodelling [90]. Conversely, elevated DBP may be more indicative of underlying vascular pathology rather than a direct cause of vascular damage and adverse outcomes [90]. Additionally, DBP and SBP are influenced by different physiological mechanisms: DBP reflects a combination of vascular resistance and arterial compliance, which exert opposing effects on its levels [90].

Our study strengthens the existing evidence base by addressing key limitations of conventional observational studies, which are often prone to confounding, and RCTs, which typically lack sufficient power to detect effects on many clinical outcomes. Unlike traditional observational designs, our MR approach uses genetic variants as instrumental variables, reducing susceptibility to confounding and reverse causation. Furthermore, the use of large-scale datasets provides the required statistical power to detect even modest effects across a broad range of clinical outcomes.

We acknowledge several study limitations. First, the validity of MR findings depends on key assumptions: instrument relevance, independence, and exclusion restriction. Through extensive sensitivity analyses, we demonstrate that the selected SNPs are relevant instruments for the target population, as they are associated with SBP/DBP during pregnancy, as well as HDP, GH, and PE. Since the correlations observed were not perfect, with the SNP effects on blood pressure being stronger among the general population than among pregnant women, the use of inflated SNP—exposure effect estimates in our analyses may have biased our MR estimates towards the null. Two factors limit our ability to draw firm inferences about true differences in SNP effects between pregnant and general populations. First, the magnitude of SNP—blood pressure associations could not be estimated with comparable precision given the large disparity in sample sizes (up to 7192 pregnant women in ALSPAC vs 1,028,980 individuals in Keaton et al.). Second, winner’s curse bias may have inflated SNP effect estimates for blood pressure traits in the general population, since the same discovery sample was used to select SNPs exceeding the conventional GWAS-significance threshold (*p* < 5e-08) [91]. With regard to the independence and exclusion restriction assumptions, our sensitivity analyses did not indicate that our main findings were substantially biased by population structure or horizontal pleiotropy, including effects from pleiotropic SNPs or fetal genetic contributions. However, we understand these two assumptions are untestable, so our MR estimates may still be biased to a certain extent.

Caution is warranted when translating our MR findings into clinical interventions aimed at reducing adverse pregnancy and perinatal outcomes via blood pressure control during pregnancy. This is because the SNPs used cannot accurately estimate the magnitude of blood pressure changes and will predict lifetime variations in blood pressure—spanning preconception, gestation, and post-pregnancy. Therefore, MR cannot inform the optimal dose/timing for therapeutic interventions. Furthermore, interpreting the magnitude of MR effect estimates requires additional, often unverifiable, assumptions, such as the monotonicity assumption, which posits that the genetic instruments influence the exposure in the same direction across all individuals. Under this assumption, we can interpret the results as the average causal effect among those people whose exposure was changed by the genetic instrument, a group that is not necessarily well defined. Finally, we are assuming linear effects of blood pressure on the outcomes, and our sample was mostly restricted to European ancestry women with low-risk pregnancies. Therefore, further evidence is needed to assess transportability of our findings to other populations. In particular, large, well-powered RCTs comparing different antihypertensive treatment regimens are essential for identifying optimal therapeutic windows and dosing strategies to prevent adverse pregnancy outcomes.

## Conclusions

In conclusion, our findings suggest that lowering maternal blood pressure is likely to have widespread benefits for maternal and offspring health, including lowering the risk of impaired fetal growth, PTB (including spontaneous PTB), need for induction of labour, GDM, and NICU admission. Additionally, our findings indicate that targeting maternal blood pressure is unlikely to affect the risk of miscarriage and stillbirth. Our evidence for perinatal depression and low Apgar at 1 and 5 min was less certain due to the modest magnitude and relatively high imprecision of the effect estimates. Overall, our results suggest the need to monitor and manage blood pressure in the population, as higher blood pressure may increase the risk of adverse perinatal outcomes.

## Supplementary Information

Additional file 1: STROBE-MR checklist.

Additional file 2: Supplementary methods.

Additional file 3. Supplementary Tables S1–S11. Table ST1–Definitions of perinatal outcomes. Table ST2–Genetic instruments for Systolic Blood Pressure in Keaton 2024 GWAS. Table ST3–Genetic instruments for Diastolic Blood Pressure in Keaton 2024 GWAS. Table ST4–Genetic instruments for Systolic Blood Pressure in Ehret 2016 GWAS (GCST006259). Table ST5–Genetic instruments for Diastolic Blood Pressure in Ehret 2016 GWAS (GCST006258). Table ST6–MR results for the genetically predicted effects of maternal blood pressure on adverse perinatal outcomes. Table ST7–Steiger directionality test for the genetically predicted effects of maternal blood pressure on adverse perinatal outcomes. Table ST8–Heterogeneity test for the genetically predicted effects of maternal blood pressure on adverse perinatal outcomes. Table ST9–MR-Egger intercept test for the genetically predicted effects of maternal blood pressure on adverse perinatal outcomes. Table ST10–Multivariable MR for the genetically predicted effects of maternal blood pressure on adverse perinatal outcomes, accounting for body mass index, height, years of education, and age at first birth. Table ST11–MR results for the genetically predicted effects of maternal blood pressure on adverse perinatal outcomes, accounting for offspring and father genotypes through weighted linear models.

Additional file 4. Supplementary Figures S1–S18. Fig. S1–Correlation plots for SBP/DBP instrumental variables and maternal blood pressure during pregnancy (i.e. first, second and third trimesters and across gestation), GH, preeclampsia and HDP. Fig. S2–Regression plots for SBP/DBP instrumental variables and maternal blood pressure during pregnancy (i.e. first, second and third trimesters and across gestation), GH, preeclampsia and HDP. Fig. S3–Study-specific and pooled Mendelian randomization estimates for the genetically predicted effects of maternal blood pressure on the primary outcomes. Fig. S4–Inverse-variance weighted Mendelian randomization estimates for genetically predicted effects of maternal blood pressure on the secondary (binary) outcomes. Fig. S5–Inverse-variance weighted Mendelian randomization estimates for genetically predicted effects of maternal blood pressure on the secondary (continuous) outcomes. Fig. S6–Study-specific and pooled Mendelian randomization estimates for the genetically predicted effects of maternal blood pressure on the secondary (binary) outcomes. Fig. S7–Study-specific and pooled Mendelian randomization estimates for the genetically predicted effects of maternal blood pressure on the secondary (continuous) outcomes. Fig. S8–Mendelian randomization estimates for genetically predicted effects of maternal blood pressure on positive control outcomes. Fig. S9–Mendelian randomization estimates for genetically predicted effects of maternal blood pressure on the secondary (binary) outcomes across different methods. Fig. S10–Mendelian randomization estimates for genetically predicted effects of maternal blood pressure on the secondary (continuous) outcomes across different methods. Fig. S11–Mendelian randomization estimates for genetically predicted effects of maternal blood pressure on determinants of perinatal health. Fig. S12–Univariable (“Inverse variance weighted”) and multivariable (“MVMR”) Mendelian randomization estimates for genetically predicted effects of maternal blood pressure on secondary (binary) outcomes. Fig. S13–Univariable and multivariable Mendelian randomization estimates for genetically predicted effects of maternal blood pressure on secondary (continuous) perinatal outcomes. Fig. S14–Mendelian randomization estimates for genetically predicted effects of maternal blood pressure on the secondary (binary) outcomes, adjusting for offspring genotype. Fig. S15–Mendelian randomization estimates for genetically predicted effects of maternal blood pressure on the secondary (continuous) outcomes, adjusting for offspring genotype. Fig. S16–Mendelian randomization estimates for genetically predicted effects of maternal blood pressure on the primary outcomes in overlapping (Keaton et al.) and non-overlapping samples (Ehret et al.). Fig. S17–Mendelian randomization estimates for genetically predicted effects of maternal blood pressure on the secondary (binary) outcomes in overlapping (Keaton et al.) and non-overlapping samples (Ehret et al.). Fig. S18–Mendelian randomization estimates for genetically predicted effects of maternal blood pressure on the secondary (continuous) outcomes in overlapping (Keaton et al.) and non-overlapping samples (Ehret et al.)

## Data Availability

ALSPAC, MoBa, BiB and UK Biobank data are available upon request. ALSPAC data were accessed under project number B3844. The ALSPAC access policy that describes the proposal process in detail including any costs associated with conducting research at ALSPAC, which may be updated from time to time and is available at: https://www.bristol.ac.uk/medialibrary/sites/alspac/documents/researchers/dataaccess/ALSPAC_Access_Policy.pd Data from the Norwegian Mother, Father and Child Cohort Study and the Medical Birth Registry of Norway used in this study are managed by the national health register holders in Norway (Norwegian Institute of public health) and can be made available to researchers, provided approval from the Regional Committees for Medical and Health Research Ethics (REC), compliance with the EU General Data Protection Regulation (GDPR) and approval from the data owners. The consent given by the participants does not open for storage of data on an individual level in repositories or journals. Researchers who want access to data sets for replication should apply through helsedata.no. Access to data sets requires approval from The Regional Committee for Medical and Health Research Ethics in Norway and an agreement with MoBa (see its website https://www.fhi.no/en/op/data-access-from-health-registries-health-studies-and-biobanks/data-access/applying-for-access-to-data/ for details). Data is available upon request from Born in Bradford: https://borninbradford.nhs.uk/research/how-to-access-data/ Researchers can apply for access to the UK Biobank data via the Access Management System (AMS) ( https://www.ukbiobank.ac.uk/enable-your-research/apply-for-access). Blood pressure GWAS data are publicly available and can be downloaded from the GWAS Catalog ( https://www.ebi.ac.uk/gwas/). Statistical code used for the two-sample MR analyses is publicly accessible as part of the “TwoSampleMR” R package: https://mrcieu.github.io/TwoSampleMR/index.html. The analytic code will be made publicly available upon publication at https://github.com/fernandam93/BP_pregnancy_MR. Data from the Norwegian Mother, Father and Child Cohort Study and the Medical Birth Registry of Norway used in this study are managed by the national health register holders in Norway (Norwegian Institute of public health) and can be made available to researchers, provided approval from the Regional Committees for Medical and Health Research Ethics (REC), compliance with the EU General Data Protection Regulation (GDPR) and approval from the data owners. The consent given by the participants does not open for storage of data on an individual level in repositories or journals. Researchers who want access to data sets for replication should apply through helsedata.no. Access to data sets requires approval from The Regional Committee for Medical and Health Research Ethics in Norway and an agreement with MoBa (see its website https://www.fhi.no/en/op/data-access-from-health-registries-health-studies-and-biobanks/data-access/applying-for-access-to-data/ for details). Data is available upon request from Born in Bradford: https://borninbradford.nhs.uk/research/how-to-access-data/ Researchers can apply for access to the UK Biobank data via the Access Management System (AMS) (https://www.ukbiobank.ac.uk/enable-your-research/apply-for-access). Blood pressure GWAS data are publicly available and can be downloaded from the GWAS Catalog (https://www.ebi.ac.uk/gwas/). Statistical code used for the two-sample MR analyses is publicly accessible as part of the “TwoSampleMR” R package: https://mrcieu.github.io/TwoSampleMR/index.html. The analytic code will be made publicly available upon publication at https://github.com/fernandam93/BP_pregnancy_MR. This is an Open Access article distributed in accordance with the terms of the Creative Commons Attribution (CC BY 4.0) license, which permits others to distribute, remix, adapt and build upon this work, for commercial use, provided the original work is properly cited. See: http://creativecommons.org/licenses/by/4.0/.

## Abbreviations

ALSPAC: Avon Longitudinal Study of Parents and Children
BiB: Born in Bradford
BMI: Body mass index
CI: Confidence interval
DBP: Diastolic blood pressure
EGG: Early growth genetics
FDR: False discovery rate
GDM: Gestational diabetes mellitus
GenDIP: Genetics of diabetes in pregnancy
GH: Gestational hypertension
GWAS: Genome-wide association study
HBW: High birth weight
HDP: Hypertensive disorders of pregnancy
ICBP: International consortium for blood pressure
InSIDE: Instrument strength independent of direct effect
InterPregGen: International Pregnancy Genetics study
IVW: Inverse variance weighted
LBW: Low birth weight
LD: Linkage disequilibrium
LGA: Large-for-gestational age
MAF: Minor allele frequency
MoBa: Norwegian Mother, Father and Child Cohort Study
MR: Mendelian randomization
MVMR: Multivariable Mendelian randomization
NICU: Neonatal intensive care unit
OR: Odds ratio
PGC: Psychiatric genomics consortium
PTB: Preterm birth
RCT: Randomised controlled trial
RR: Risk ratio
SBP: Systolic blood pressure
SE: Standard error
SGA: Small-for-gestational age
SNP: Single nucleotide polymorphism
STROBE-MR: Strengthening the Reporting of Observational Studies in Epidemiology using Mendelian Randomization
UKB: UK Biobank
WLM: Weighted linear model
ZEMPA: ZEro modal pleiotropy assumption
